# Fast Dissolving of Ferulic Acid via Electrospun Ternary Amorphous Composites Produced by a Coaxial Process

**DOI:** 10.3390/pharmaceutics10030115

**Published:** 2018-08-02

**Authors:** Weidong Huang, Yaoyao Yang, Biwei Zhao, Gangqiang Liang, Shiwei Liu, Xian-Li Liu, Deng-Guang Yu

**Affiliations:** 1 School of Chemistry and Chemical Engineering, Hubei Polytechnic University, Huangshi 435003, China; neweydong@hbpu.edu.cn; 2 Hubei Key Laboratory of Mine Environmental Pollution Control and Remediation, Hubei Polytechnic University, Huangshi 435003, China; 3 School of Materials Science and Engineering, University of Shanghai for Science and Technology, Shanghai 200093, China; yyyang@usst.edu.cn (Y.Y.); 1526410303@st.usst.edu.cn (B.Z.); 1526410214@st.usst.edu.cn (G.L.); 1526410109@st.usst.edu.cn (S.L.)

**Keywords:** amorphous composite, coaxial electrospinning, fast dissolution, insoluble drug, solid dispersion

## Abstract

Enhancing the dissolution of insoluble active ingredients comprises one of the most important issues in the pharmaceutical and biomaterial fields. Here, a third generation solid dispersion (3rd SD) of ferulic acid was designed and fabricated by a modified coaxial electrospinning process. A traditional second generation SD (2nd SD) was also prepared by common one-fluid blending electrospinning and was used as a control. With poly(vinyl alcohol) as the fiber matrix and polyvinylpyrrolidone K10 as an additive in the 3rd SDs, the two electrospinning processes were investigated. The prepared 2nd and 3rd SDs were subjected to a series of characterizations, including X-ray diffraction (XRD), scanning electron microscope (SEM), hydrophilicity and in vitro drug dissolving experiments. The results demonstrate that both SDs were monolithic nanocomposites and that the drugs were amorphously distributed within the matrix. However, the 3rd SDs had better morphology with smaller size, narrower size distribution, and smaller water contact angles than the 2nd SDs. Dissolution tests verified that the 3rd SDs could release their loaded cargoes within 60 s, which was over three times faster than the 2nd SDs. Therefore, a combined strategy based on the modified coaxial electrospinning and the logical selections of drug carriers is demonstrated for creating advanced biomaterials.

## 1. Introduction

Many drugs are insoluble or poorly water soluble, and improving their dissolution is one of the central challenges in pharmaceutics [1,2,3,4]. Among the different kinds of strategies that have been broadly investigated to resolve this problem, drug solid dispersions (SDs) are some of the most promising ways with many commercial products [5,6,7,8,9]. New methods for creating new kinds of high-performance SDs are always desired and are thus becoming a rapidly developing branch of pharmaceutical technologies [10,11,12,13,14,15]. 

**Figure 1:**
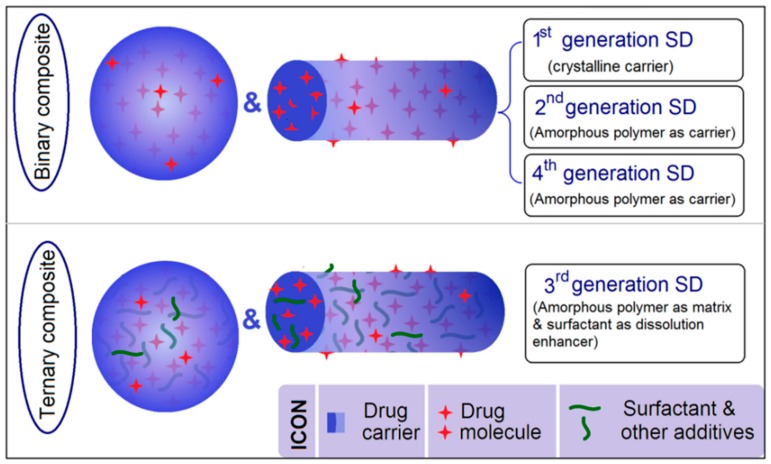
A diagram showing the development of solid dispersions (SDs) from the initial first generation to the third and fourth generations.

Over the past half century, first generation SDs (1st SDs) have progressed to second (2nd SDs) and third generation SDs (3rd SDs) [16,17]. Figure 1 is a schematic of the differences in their characteristics. Often, 1st and 2nd SDs are binary systems with the drug distributed in carriers. In 1st SDs, carriers are mainly crystalline pharmaceutical excipients; in 2nd SDs, amorphous polymers are frequently used as host polymers. The concept of 3rd SDs has gained the interest of researchers in related fields [18]. This new type of SD is typically a ternary or quaternary system with one or two additives (such as surfactants and other polymeric excipients) that, together with the drug and host polymer, are combined to exert a synergistic effect on drug-dissolution improvement [17,18]. The polymer-based SDs are amorphous composites. When an amorphous polymer is exploited not only for drug dissolution but also for controlling drug release, the SD is often termed a 4th generation SD [19,20]. 

Considering the present nano-era, SDs are inevitably advancing to nanoproduction because a fine size always means large surface area for functional performance. Among different pharmaceutical nanotechnologies, electrospinning is distinct from others because of its high effectiveness, easy implementation, and low cost for creating polymer nanofibers [21,22,23,24,25,26,27,28]. Electrospinning and also electrospraying are simple, one-step, “top-down” electrohydrodynamic atomization (EHDA) processes that have quickly developed from the traditional one-fluid blending process to two-fluid (side-by-side and coaxial) processes, and also to tri-axial processes [29,30,31,32,33]. Some studies have demonstrated that single-fluid EHDA and traditional coaxial EHDA are powerful tools for creating SDs [34,35].

For SDs produced from a co-dissolving solution using the one-fluid blending process, several amorphous water-soluble polymers have been investigated. These polymers include poly(ethylene oxide), polyvinylpyrrolidone (PVP), poly(vinyl alcohol) (PVA), gelatin, and some other natural products, e.g., water-soluble polysaccharides [36,37]. Among them, PVA has wide-ranging applications in the pharmaceutical, cosmetic, food, medical and packaging industries. In the field of biomaterials, PVA gels are exploited as drug carriers directly or in the form of particles added to other carriers for tablet formulation. However, these studies have focused only on drug-sustained release; cross-linking is often carried out for creating insoluble nonwoven mats [36]. For SDs, the role of the carrier in forming SDs is very important [38,39]. PVA, as a highly water soluble, highly biocompatible and nontoxic polymer, is a good candidate carrier for promoting drug dissolution [36,37]. 

Ferulic acid (FA), an abundant polyphenol in maize bran and vegetables, was utilized as a model of a poorly water-soluble drug. It has been explored for a wide variety of potential applications such as age-related diseases, cancer, cardiovascular diseases and diabetes [40]. However, its poor solubility has greatly limited its oral bioavailability [41]. Thus, based on our previous work on the fabrication of SD using traditional coaxial electrospinning [42], here, the usage of advanced modified coaxial electrospinning to prepare 3rd SDs was investigated for the first time.

## 2. Materials and Methods 

### 2.1. Materials

PVA (*M*_w_ = 170,000 g/mol; 88% hydrolyzed) was bought from Shanghai Meimengjia Chemical Co., Ltd. (Shanghai, China). FA (purity > 99%) was purchased from Shanghai Rong-Chuang Biotechnology Co., Ltd. (Shanghai, China). PVP K10 with a molecular weight of 10,000 g/mol was obtained from Sigma-Aldrich Co., Ltd. (Shanghai, China). Anhydrous ethanol and methylene blue were bought from Shanghai Chemical Reagents Co., Ltd. (Shanghai, China). 

### 2.2. Electrospinning

The electrospinning system had the following four parts: a power supply (ZGF60kV-2mA, Wuhan Hua-Tian Corp., Wuhan, China), two fluid drivers (KDS100 and KDS200, Cole-Parmer, Vernon Hills, IL, USA), a collector and a homemade concentric spinneret. After some optimization experiments, the preparation conditions were determined as follows: an applied voltage of 14 kV, a spinneret-collector distance of 15 cm, and a fixed core fluid flow rate of 1.0 mL/h. The environmental temperature and humidity were 21 ± 4 °C and 51 ± 5%, respectively. The properties of the working fluids, including conductivity, surface tension and viscosity were measured using a conductivity meter (DDS-11, Shanghai Rex Co-perfect Instrument Co., Ltd., Shanghai, China), a surface tension tensiometer (BZY-1, Shanghai Hengping Instrument & Meter Factory, Shanghai, China), and a rotary viscometer (NDJ 279, Machinery & Electronic Factory of Tongji University, Shanghai, China), respectively. All the experiments were repeated three times. 

### 2.3. Morphology

The nanofibers’ morphologies were studied with the aid of a Quanta FEG450 scanning electron microscope (FE-SEM; FEI Corporation, Hillsboro, OR, USA). Samples were platinum sputter-coated under a nitrogen atmosphere for 100 s prior to visualization. Fiber diameters were calculated using the ImageJ software (National Institutes of Health, Bethesda, MD, USA) to measure the fibers at 100 different points. 

### 2.4. Physical Form and Compatibility

X-ray diffraction (XRD) patterns were collected on a D/Max-BR diffractometer (Rigaku, Tokyo, Japan) over the 2θ range 5 to 60°. The instrument was supplied with Cu Kα radiation at 40 mV and 30 mA. Differential scanning calorimetry (DSC) was carried out using an MDSC 2910 differential scanning calorimeter (TA Instruments Co., New Castle, DE, USA). Sealed samples were heated at 10 °C·min^−1^ from 21 to 250 °C. The nitrogen gas flow rate was kept at 40 mL·min^−1^. Fourier transform infrared (FTIR) spectroscopy was carried out on a Nicolet-Nexus 670 FTIR spectrometer (Nicolet Instrument Corporation, Madison, WI, USA) at a range of 500 to 4000 cm^–1^ and a resolution of 2 cm^−1^.

### 2.5. Property and Functional Performances

A DSA100 drop analysis instrument (Krüss GmbH, Hamburg, Germany) was exploited to measure the surface contact angle (WCA) of nanofiber mats. Distilled water droplets (3 µL) were placed onto the sample’s surface. Six different regions of each surface were measured, and the obtained data were averaged. 

FA has a maximum UV absorbance at λ_max_ = 321 nm and a shoulder at 278 nm. A calibration curve was thus constructed at 321 nm with the aid of a Lambda 750S spectrophotometer (Perkin Elmer, Waltham, MA, USA). This took the form *C* = 17.74*A* + 0.14 (R^2^ = 0.9996), where *C* is the concentration of FA (μg/mL) and *A* is the absorbance (linear range: 1–20 μg/mL).

Drug release was quantified following the Chinese Pharmacopoeia Method II (a paddle method) on RCZ-8A dissolution apparatus (Tianjin University Radio Factory, Tianjin, China) at 50 rpm and 37 °C. Then, 30 mg of each sample was placed into 600 mL of phosphate buffered saline (PBS, pH = 7.0, 0.1 M). At predetermined time points, 5 mL aliquots were withdrawn, and 5 mL of fresh preheated PBS was added to maintain a constant volume. The absorbance of the aliquots at λ_max_ = 321 nm was used to determine the amount of FA released at each time point (with suitable dilution performed where required, to ensure the absorbance lay within the calibration range). Dissolution tests for each sample were performed six times. 

### 2.6. Statistical Method

All experiments for statistical analysis were repeated with a minimum of *n* = 6. Statistical analysis was performed using two-way analysis of variance (ANOVA). Statistically significant values were defined at *α* = 0.05.

## 3. Results and Discussion

### 3.1. One System but Two Different Electrospinning Processes 

**Figure 2:**
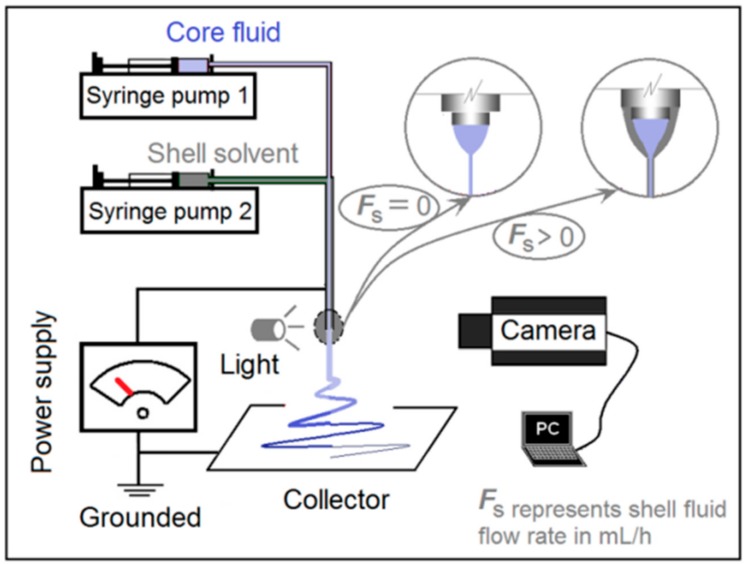
Schematic of the electrospinning system.

**Figure 3:**
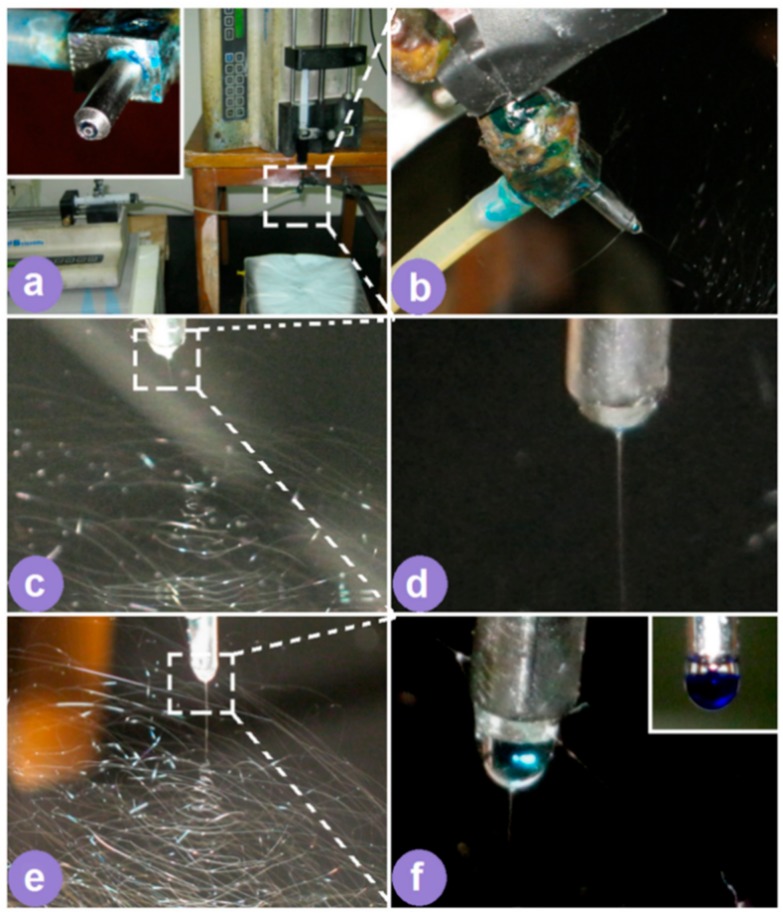
Two electrospinning processes. (**a**) An image of the working system; the inset is the concentric spinneret. (**b**) The connection between the spinneret and the power supply. (**c**) An image of the one-fluid electrospinning process. (**d**) An enlarged image of the Taylor cone in a single-fluid process. (**e**) An image of the coaxial process. (**f**) An enlarged image of the Taylor cone in a two-fluid coaxial process.

Figure 2 is a schematic of the homemade electrospinning system that can be used to implement both one-fluid blending electrospinning and two-fluid coaxial processes. When the sheath fluid flow rate (*F*s) was turned off, i.e., *F*s = 0 mL/h, it was a typical one-fluid blending spinning process; when *F*s > 0 mL/h, it became a two-fluid coaxial spinning process. In the literature, almost all single-fluid electrospinning processes are carried out using a metal capillary as a spinneret, and all double coaxial processes are carried out using a concentric spinneret [17,32]. In the present study, one system with a key component (concentric spinneret) was used for both processes. An image of the working system is shown in Figure 3a. The system consisted of two syringe pumps, a collector, a concentric spinneret (inset of Figure 3a), and a power supply. The power supply was connected with the spinneret through an crocodile clip (Figure 3b). 

In the preparation of 2nd SDs, a blending solution composed of 13% (*w*/*v*) PVA and 2.0% (*w*/*v*) FA in 50% (*v*/*v*) aqueous ethanol was used as the working fluid for the one-fluid blending electrospinning. In the preparation of 3rd SDs using the modified coaxial process, 50% (*v/v*) aqueous ethanol was used as the sheath fluid. A co-dissolving solution composed of 13% (*w*/*v*) PVA, 2% (*w*/*v*) PVP K10, and 2% (*w*/*v*) FA in 50% (*v*/*v*) aqueous ethanol was exploited as the core working liquid. For observing the experimental processes, methylene blue (5 ng/mL in 50% (*v/v*) aqueous ethanol) was mixed into the core solutions. Details for the preparation are included in Table 1.

Figure 3c shows a single-fluid electrospinning process, where a Taylor cone (Figure 3d) was adjacent to a straight fluid jet and to bending and whipping loops for drawing the fluid jets. Similarly, when coaxial electrospinning was carried out (Figure 3e), a straight fluid jet was ejected out from a compound Taylor cone (Figure 3f), which was followed by a series of enlarged loops. The inset of Figure 3f shows an initial droplet before applying a high voltage, suggesting an easy diffusion of the methylene blue dye from the core solution to the sheath solvent in the static state. 

As shown in Table 2, the addition of PVP K10 into the PVA and FA co-dissolving solutions increased their viscosity and slightly elevated their surface tension and conductivity. These changes had little influence on their filament-forming property. However, when the sheath solvent mixture was exploited to surround the core spinnable fluid, it generated a significant influence on the working processes. The coaxial working process was initiated more easily, the Taylor cone was rounder, and the straight fluid jet was shorter than the cases in the single-fluid process, which is obvious from a comparison of Figure 3c,d with Figure 3e,f. In the electrical field, the electronic energy always gathered on the surface of fluid jets. The small viscosity and surface tension of the sheath solvent mixture (Table 2) played their positive roles for a stable and continuous electrospinning process, offsetting the negative influences of a small conductivity.

### 3.2. Morphology 

**Figure 4:**
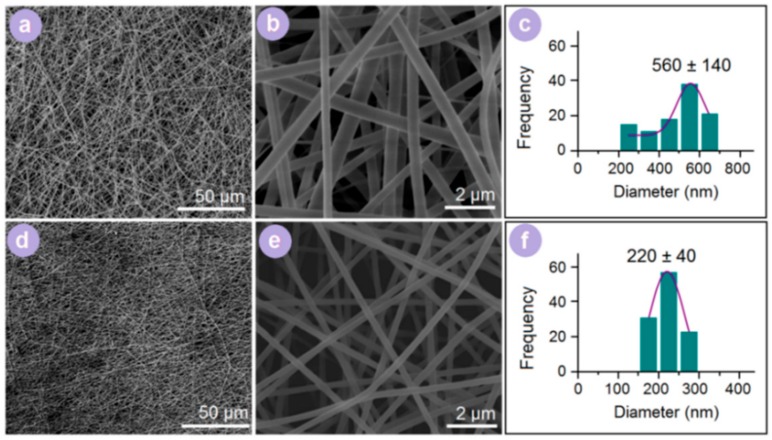
Scanning electron microscope (SEM) images of electrospun nanofibers and their size distributions. (**a**,**b**) Morphology of 2nd SDs with different magnifications. (**c**) Diameter distribution of 2nd SDs. (**d**,**e**) Morphology of 3rd SDs with different magnifications. (**f**) Diameter distribution of 3rd SDs.

One-fluid and modified coaxial processes were both able to create composite nanofibers that were assessed by SEM. All of them had a linear morphology without any discernible bead or spindle. However, the differences in the two types of nanofibers were significant. As shown in Figure 4a,b, the 2nd SDs had an average diameter of 560 ± 140 nm (Figure 4c). The 3rd SDs from the modified coaxial process (Figure 4d,e) had an average diameter of 220 ± 40 nm (Figure 4f), meaning a higher quality than the 2nd SDs in terms of a smaller diameter and more concentrated size distribution. The sheath solvents in the modified coaxial process retained a stable and robust spinning process and enabled a longer time drawing on the fluid jets to further downsize the nanofibers. 

### 3.3. Physical Forms

**Figure 5:**
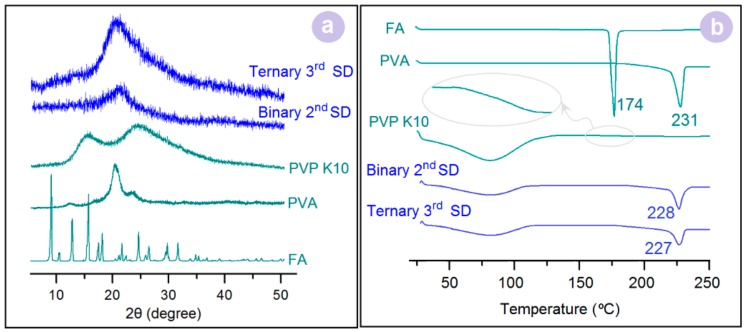
(**a**) XRD patterns; (**b**) DSC thermograms.

The X-ray powder diffraction (XRD) patterns of the crude materials (FA, PVA, and PVP K10), 2nd SDs, and 3rd SDs are shown in Figure 5a. The numerous sharp peaks in the patterns of FA suggest that the raw FA particles were crystalline. In sharp contrast, the two halos in the patterns of PVP K10 suggest that the raw PVP particles were an amorphous polymer matrix. The semi-crystalline, hydrophilic nature of PVA was demonstrated by the single blunt peak. However, both binary and ternary SDs had no sharp peak of the drug and had only one clear hump. These results suggest that both of them were monolithic and amorphous regardless of the two or three components present within the nanofibers. DSC thermograms of the crude powders (FA, PVA, and PVP K10), 2nd SDs, and 3rd SDs are shown in Figure 5b. These data concur with the XRD results. FA and PVA each had a single melting point at 174 and 231 °C, respectively. PVP had a blunt dehydrated endothermic peak before 100 °C, followed by a small conversion slope from the glass state to a rubber state around 170 °C (the inset of Figure 5b). However, in the curves of 2nd SDs and 3rd SDs, no peaks of FA could be detected along with the peaks from PVP and PVA, suggesting that FA was completely converted into an amorphous state. Meanwhile, the drug FA generated some plasticization effects on PVA, moving the melting points from 231 °C to 228 °C and 227 °C for 2nd SDs and 3rd SDs, respectively. 

**Figure 6:**
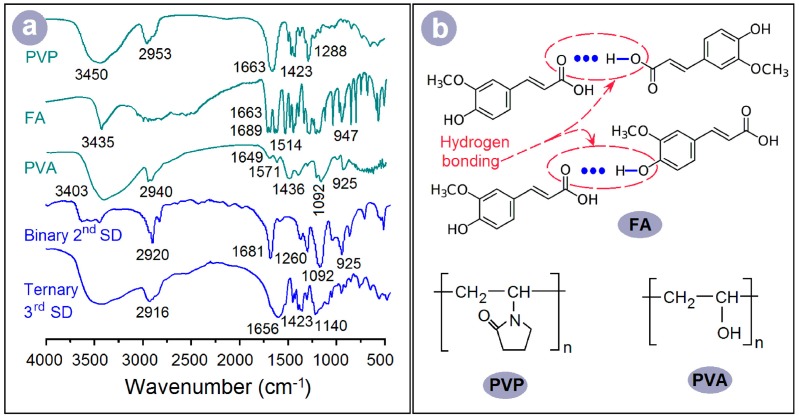
(**a**) FTIR spectra; (**b**) The molecular formula of the raw materials, ferulic acid (FA), polyvinylpyrrolidone (PVP), and poly(vinyl alcohol) (PVA).

Shown in Figure 6a are the FTIR spectra of the crude powders (FA, PVA, and PVP K10) and their binary 2nd SDs and ternary 3rd SDs. Their molecular formulae are shown in Figure 6b. The FTIR spectra of FA powders have a series of sharp peaks, such as 1689 and 1663 cm^−1^ (Figure 6a). These peaks should result from the stretching vibration of C=O groups, giving a hint that they were in a different crystal lattice (Figure 6b). FA molecules have both OH and C=O groups, PVA molecules have numerous OH groups and PVP molecules have numerous C=O groups. When electrospun into binary 2nd SDs or ternary 3rd SDs, they were compatible because of the favorable secondary interactions between the drug and the polymers. 

In the spectra of binary 2nd SDs, there is, only one, sharp peak at 1681 cm^−1^ due to the stretching vibration of C=O groups, suggesting that FA lost its original crystal state in the binary nanocomposites with PVA. In the spectra of binary 3rd SDs, the large peak at 1656 cm^−1^ was a combined result from the stretching vibration of C=O groups both in the FA and also PVP molecules, similarly suggesting that FA was amorphous in the ternary nanocomposites. Meanwhile, the intensities of several sharp peaks in the fingerprint region of FA spectra were greatly decreased or had totally disappeared, giving hints about the formation of amorphous composites between FA and PVA in the binary 2nd SDs and between PVA and PVP in the ternary 3rd SDs. 

### 3.4. Property and Functional Performance

**Figure 7:**
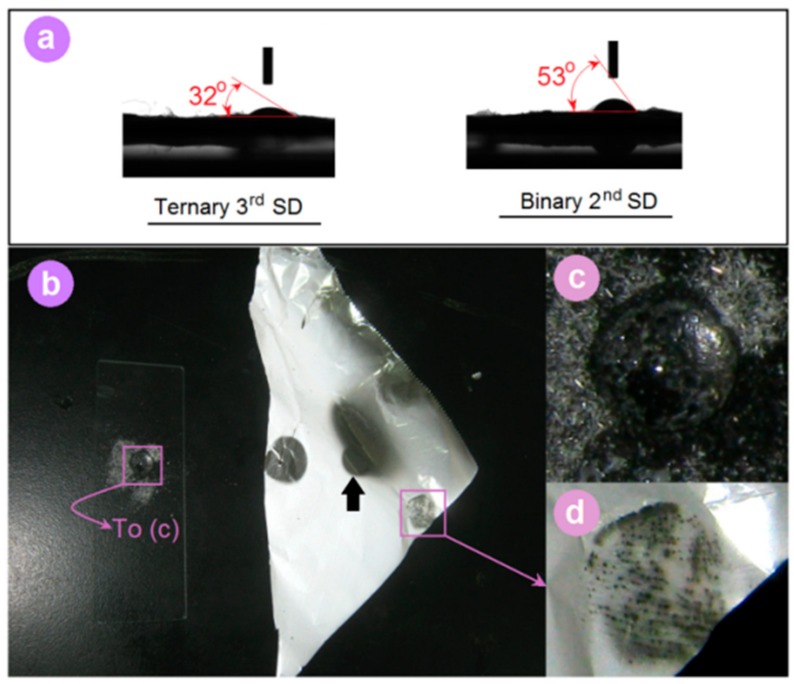
(**a**) Water contact angles (WCA). (**b**) Digital images when water droplet was dripped on the FA powders and the electrospun ternary SDs; (**c**) An enlarged image of the drop of water on the powder; (**d**) A fingermark on the electrospun nanofibers due to moisture.

PVA is a highly water-soluble polymer. Both types of electrospun nanofibers were highly hydrophilic. The water droplets rapidly receded after they were placed on the fibers’ surfaces. Thus, to differentiate the hydrophilicity of SDs, WCA was recorded after a water droplet was placed on their surface for 2 s. Average Water Contact Angle (WCA) values (*n* = 6) and typical images are shown in Figure 7a. The 3rd SDs had an average value of 31.7° ± 5.2°, smaller than that of 2nd SDs, which had an average value of 53.3° ± 7.8°. This difference suggests that the 3rd SDs had better hydrophilicity than their counterpart due to the third additive PVP K10 and also their smaller diameter. 

Figure 7b shows an intuitive impression on the hydrophilic properties of the raw FA powders and the FA-loaded ternary nanofiber film. FA is a poorly water-soluble drug. Thus, the droplet of water stood on the powders without any discernible dissolution (Figure 7c). In sharp contrast, the FA-loaded film was dissolved at once, as indicated by the arrow in Figure 7b. When a finger was pressed on the fiber film, a clear fingermark appeared because of the moisture (Figure 7d). These phenomena demonstrate that the electrospun nanofibers had fine hydrophilicity, and the ternary SDs were better than the binary SDs.

**Figure 8:**
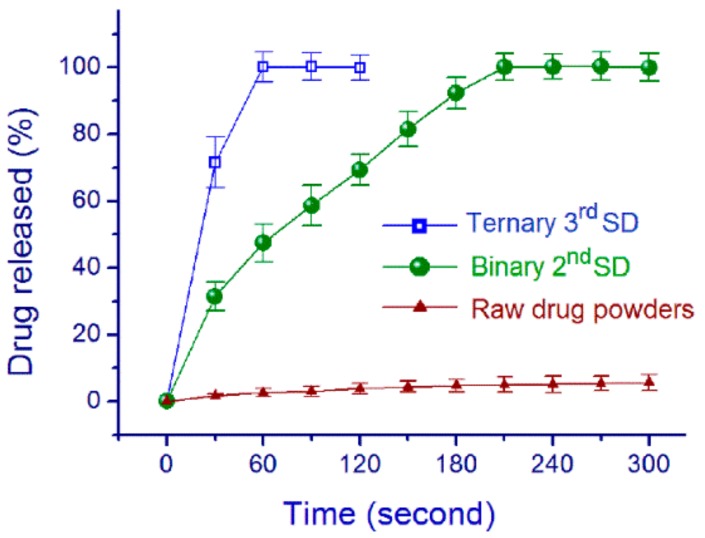
Drug-release profiles of the raw FA powders, the 2nd SDs and 3rd SDs.

Figure 8 shows the in vitro drug release profiles of raw FA powders and the 2nd and 3rd SDs. After 5 min, only 5.7 ± 2.4% of the raw FA powders were freed into the dissolution media. The 2nd SDs released all their cargoes after 220 s, whereas the 3rd SDs needed only 60 s. Although both of them were amorphous and monolithic electrospun nanofibers, their functional applications in enhancing drug dissolution significantly differed. This could be attributed to the fact that the 3rd SDs had better hydrophilicity than their counterparts and they also had a smaller diameter. 

**Figure 9:**
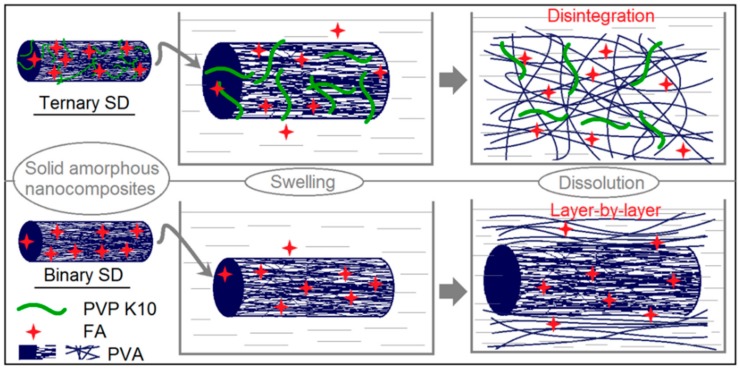
A diagram comparing the dissolution process of the 2nd SDs and 3rd SDs.

Figure 9 is a schematic of the nanostructures of the two SDs and their dissolution processes. The 2nd and 3rd SDs had average diameters of 560 and 220 nm, respectively. For the nanofibers with a cylindrical shape, their volume (*V*) can be achieved according to the equation of *V* = *r*^2^π*L,* where *L* and *r* are the length of fiber and the radius of its cross-section. The nanofiber’s surface area (*S*) can be obtained by the equation *S* = 2π*rL.* Based on the same volume of nanofibers of 2nd SDs (*V*_1_ = *r*_1_^2^π*L*_1_) and 3rd SDs (*V*_4_ = *r*_4_^2^π*L*_4_), the following relationship can be deduced: *r*_2_^2^π*L*_2_ = *r*_3_^2^π*L*_3_, i.e., *L*_3_/*L*_2_ = *r*_2_^2^/*r*_3_^2^. Thus, the surface area ratio of 3rd SDs to 2nd SDs can be calculated as follows:

*S*_3_/*S*_2_ = (2π*r*_3_*L*_3_)/(2π*r*_2_*L*_2_)

= *r_3_*/*r*_2_·*L*_3_/*L*_2_

= *r_3_*/*r*_2_·*r*_2_^2^/*r*_3_^2^ = *r*_2_/*r*_3_

= 560/220 = 2.5

This is to say that the reduction in nanofiber diameter from 560 nm for 2nd SDs to 220 nm for 3rd SDs resulted in a significant increase in the fiber’s total surface area by 2.5-fold. The smaller diameter meant double nano effects for drug dissolution, one was a larger surface area and the other was a shorter distance for the water/drug molecules to diffuse in/out of the bulk dissolution media.

However, the most important reason should be the presence of PVP in the PVA matrix, which effectively promoted the disintegration of PVA gels owing to PVP’s relatively small molecular weight, high solubility in water, and high permeation ability. The dissolution of a polymer always experiences water absorbance, swelling, and disentanglement of the polymer chains before the polymeric molecules enter the dissolution media. Shown in Figure 9, the PVP K10 molecules completely revised the dissolution processes of PVA molecules. It made the 3rd SDs nanofibers swell more quickly than the 2nd SDs nanofibers. Meanwhile, it promoted the 3rd SDs to dissolve in a disintegration manner, getting rid of the traditional layer-by-layer dissolution model that happens in the 2nd SDs. Certainly, during these processes, the water and also the drug molecules were able to diffuse more easily into and out of the gel regions formed by the PVA matrix within the 3rd SDs than the 2nd SDs. 

The generation of electrospun nanofibers on a large scale is drawing increasing attention in both the scientific research and industrial fields [43]. The ternary amorphous nanocomposites hold great promises for further developing fast dissolution drug delivery systems, which is popular for the administration of many active ingredients [44]. In future, these medicated nanofibers can be converted into different kinds of dosage forms, such as tablets, membranes, and also capsules [45]. 

## 4. Conclusions

We successfully prepared 2nd and 3rd SDs by a one-fluid blending electrospinning process and a two-fluid coaxial process, respectively. Both processes were conducted smoothly and continuously, and the resulting nanofibers had fine linear morphology without any discernible bead or spindle. Although XRD patterns demonstrated that both SD types were monolithic and amorphous nanoproducts, 3rd SDs showed over three times faster drug-release rate than 2nd SDs. The added third component PVP, the smaller diameter and corresponding larger surface, the shorter water/drug molecule diffusion distance, and the improved hydrophilicity exerted a combined effect to result in the better performance of 3rd SDs. This study has provided a new protocol for designing and developing new kinds of functional biomaterials as an alternative solution to the problem of insoluble drugs.

## Figures and Tables

**Table 1:** Parameters for solid dispersion (SD) preparations.

No.	Electrospinning	Sheath Fluid	Core Fluid	Flow Rate (mL/h)	
				Sheath	Core
2nd	Blending	--	13% (*w*/*v*) PVA and 2% (*w*/*v*) FA in 50% (*v*/*v*) aqueous ethanol	--	1.0
3rd	Modified coaxial process	50% (*v*/*v*) aqueous ethanol	13% (*w*/*v*) PVA, 2% (*w*/*v*) PVP K10 and 2% (*w*/*v*) FA in 50% (*v*/*v*) aqueous ethanol	0.2	1.0

Abbreviation: PVP = polyvinylpyrrolidone, PVA = poly(vinyl alcohol), and FA = Ferulic acid.

**Table 2:** The properties of the three working fluids during the electrospinning processes (*n* = 6).

Electrospinning Working Fluid	Viscosity	Surface Tension	Conductivity
	(cp)	(N·m^−1^ × 10^−3^)	(μS·cm^−1^)
Fluid for the blending process	212.4 ± 4.5	87.6 ± 1.2	57.4 ± 0.5
Core fluid of the coaxial process	343.7 ± 6.8	93.3 ± 0.7	57.8 ± 0.5
Sheath fluid of the coaxial process	2.87 ± 0.04	27.5 ± 0.4	0.87 ± 0.02
